# Outcomes of popliteal artery injuries in a level 1 trauma centre: a 6-year review

**DOI:** 10.1007/s00068-024-02691-9

**Published:** 2025-01-24

**Authors:** Kewen van Rensburg, Wilme Steyn, Ismail Cassimjee, Maeyane Stephens Moeng

**Affiliations:** 1 Department of Surgery, Division of Vascular Surgery, Charlotte-Maxeke Johannesburg Academic Hospital, University of Witwatersrand, Johannesburg, South Africa; 2 Department of Surgery, Division of Trauma Surgery, Charlotte-Maxeke Johannesburg Academic Hospital, University of Witwatersrand, Johannesburg, South Africa

**Keywords:** Popliteal artery Injury, Trauma, Low and middle income countries (LMIC), Amputation, Delay

## Abstract

**Purpose:**

To determine modifiable and non-modifiable factors contributing to limb loss in PAI the relevance and accuracy of published scoring systems for PAI within a South African State hospital.

**Methodology:**

Retrospective review of patients (> 18 years) with PAI, presenting to CMJAH trauma unit from 1 January 2017 to 31 December 2022.

**Results:**

Sixty-four patient records were analysed. Thirty (46.9%) had blunt trauma and thirty-four (53.1%) had penetrating trauma. Gunshot wounds (GSW) were the most common mechanism of injury (MOI). Blunt PAI had a 40% amputation rate and penetrating trauma, 33.3%. Forty-seven (73.4%) had a delay to surgery of > 6 h. The mean time to arrival at our emergency department was 478 min, and the mean time from arrival to surgery was 368 min (total delay of 838 min). The primary amputation rate was 28.6%, and 63.5% had successful limb salvage surgeries. The secondary amputation rate was 7.8%.

**Conclusion:**

Compared to international literature, our rate of primary amputation is high (10% vs. 28.8%) and prolonged ischaemia is the likely cause. Only 17 (26.6%) patients presented before 6 h. Of the 45 patients that had an attempt at revascularisation, 7.8% had a secondary amputation. Thus, despite prolonged ischaemia, revascularisation should be attempted in patients with at least two viable compartments on fasciotomy. The MESS and POPSAVEIT scoring systems should not be relied on in patients with delayed presentations. Strengthening referral triage for suspected PAI to Level 1 Trauma centres directly will decrease the delays and likely improve the outcomes.

## Introduction

Globally, Popliteal Artery Injuries (PAI) are considered uncommon. Concomitant bony or soft tissue injuries occur frequently with PAIs and this results in high rates of morbidity, limb loss, functional disability and mortality [1]. Hafez et al., reported that popliteal artery injuries carried the highest rate of limb loss (25.6%) in all lower limb arterial injuries [2]. South Africa is a low-middle income country and vascular injuries such as PAIs are a major contributor to limb loss [3] and although uncommon, they place a significant burden on scarce resources. Furthermore, PAI specifically, is an injury that mainly afflicts people in their young adult life during their most economically productive years, and this further impacts the broader society.

Blunt trauma is considered to be the leading cause of PAI’s, accounting for up to 75% of all cases of PAI’s worldwide [1, 4–6]. They are thought to have higher rates of amputations when compared to penetrating trauma and South Africa has high rates of penetrating trauma. A previous study performed at Chris Hani Baragwanath Academic Hospital in Soweto reflecting on this higher incidence of penetrating trauma, found an overall amputation rate of 14% [7], however, this study did not include PAI’s secondary to blunt trauma. In comparison, data from the United States of America National Trauma Data Bank from 2003 reported a 9% amputation rate of penetrating PAI involving combined arterial, venous and nerve injury [1].

One cannot examine PAIs without looking at the impact of delays to surgery on overall outcomes. The initial 6-hour period was first described in 1949 as the absolute limit for successful revascularization and limb salvage, and this is the “golden period”, and by decreasing ischaemic time from arrival to restoration of perfusion, limb salvage will likely be improved [8]. Prolonged delays in surgical time have been demonstrated by others to adversely affect limb salvage rates with some studies finding a 65% increased risk of primary amputation for every 6 h delay to surgery [9]. A study performed in Cape Town, concluded that the most significant factors associated with their high amputation rate (37.5%) was an ischemic time longer than seven hours [10].

The mangled extremity severity score (MESS) was developed in 1990 to determine the likelihood of successful limb salvage in patients with mangled extremities [11]. However, the validity of this scoring system has subsequently been questioned [12, 13]. A team-approach involving experienced surgeons is recommended for decision making regarding amputation as the MESS scoring system has a limited predictive value in PAI [14]. The POPSAVEIT scoring system is a contemporary scoring system which was developed in response to the lack of other validated scoring systems in predicting amputation risks in PAI’s [15]. This scoring system aims to risk stratify patients in a pre-operative setting. A high score (of ≥3) had a reported sensitivity of 89% and a specificity of 47% for amputation. However, it is important to note, that even in patients with a maximum score of 5, the predicted risk of amputation is still less than 50%. The MESS and POPSAVEIT scoring systems have routinely been used as a method to predict the risk of amputation in patients with PAI’s. However, the accuracy of these scoring systems in resource constrained settings is unknown.

We performed a retrospective study of 64 PAIs in a tertiary government funded Level 1 trauma referral centre in Johannesburg. Although the literature is replete with case series of PAIs, there is a paucity of information from resource constrained environments, especially where vascular surgical emergency services are provided by vascular surgeons and not general surgeons.

The type of surgeon performing the vascular repair, either trauma surgeon or vascular surgeon, as demonstrated by Krüger et al. also plays a role in the outcome of these patients with the findings of improved outcomes in patients operated on by vascular surgeons [16].

Thus, it was our objective to interrogate factors that contribute to poorer outcomes in PAI’s, aiming to determine if there were modifiable factors within a South African setting. Also, we aimed to assess the validity of universally accepted scoring systems to patients in the South African setting.

## Methodology

We performed a retrospective analysis of data from patients seen at Charlotte-Maxeke Johannesburg Academic Hospital (CMJAH) trauma unit over 6 years (from 1 January 2017 to 31 December 2022). Data was collected from MediBank (this is an electronic trauma database, captured prospectively), patient files, as well as from the online discharge database of patients.

**Fig. 1:**
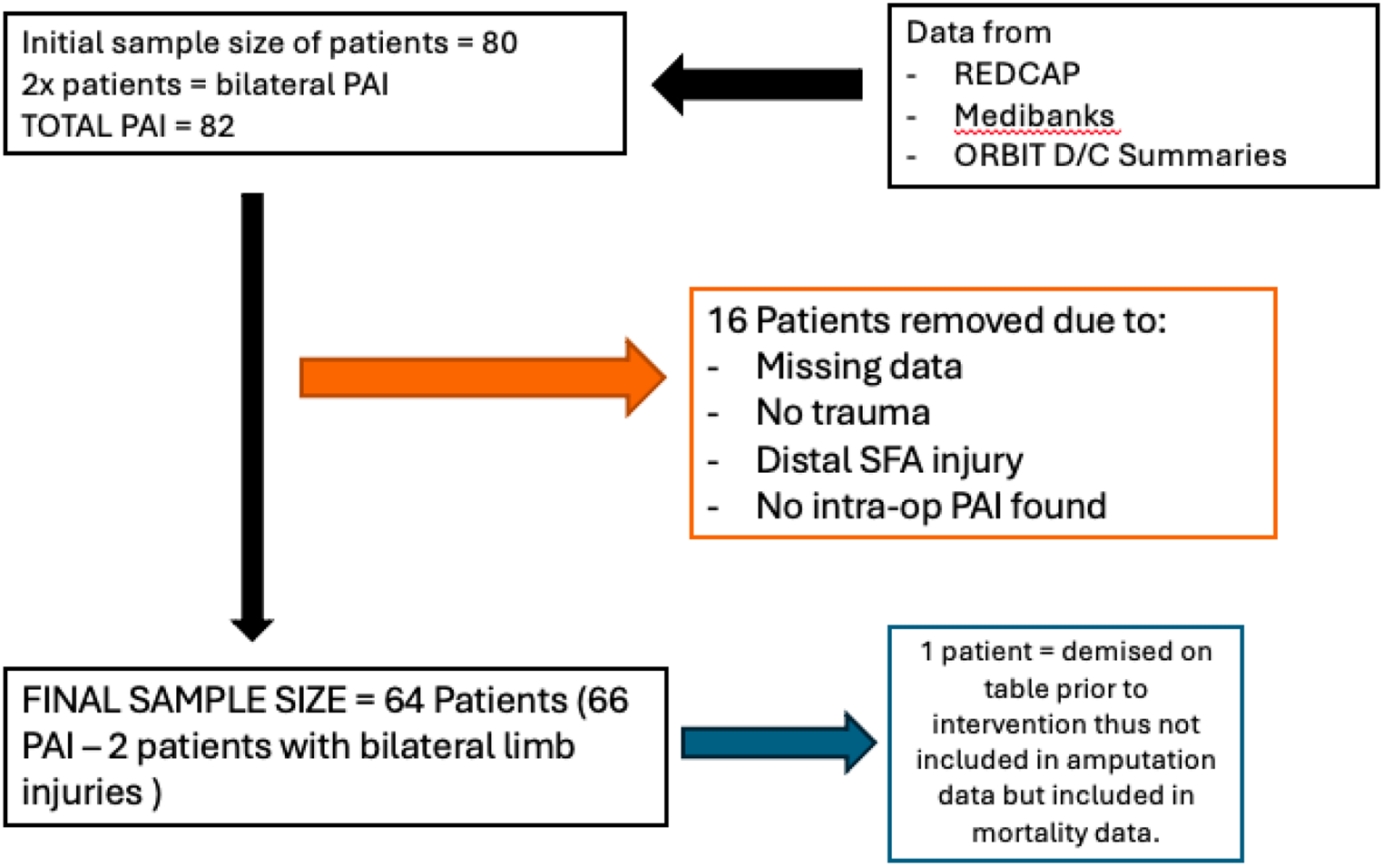
Consort diagram of study population and exclusions

In our study population, we included all patients admitted and managed by the CMJAH trauma unit older than 18 years of age, during the specified time. From our initial data collection, we had identified 80 patients suitable for our study. From this initial number, 16 patients had to be excluded as depicted in Fig. 1. Of note, 1 patient died on table prior to any surgical intervention and was included as a mortality. To clarify, information on the outcome variable of amputation will have data on 63 patients while information on the outcome variable of mortality, has data for all 64 patients.

The Charlotte-Maxeke Johannesburg Academic hospital is a level 1 Trauma referral centre, one of only 2 level 1 centres in the public hospital sector of Johannesburg, serving a population of 7 million people. Patients injured in the local vicinity are transferred directly to the hospital and those in the drainage areas of referral hospitals are taken to their nearest hospital first prior to transfer.

All patients are assessed by the trauma team on arrival as per Advanced Trauma Life Support (ATLS) protocols. Open wounds are irrigated and dressed, and displaced fractures are reduced prior to imaging. Suspected PAI injuries are investigated with a CT angiogram if they are haemodynamically stable. Haemodynamically unstable patients are transferred immediately to the operating room (OR) and are investigated with on table angiography. Once confirmed, PAIs are referred to the vascular surgical team for further assessment and treatment. Patients with Rutherford 1 ischaemia undergo a popliteal artery repair without a fasciotomy, those with Rutherford 2 ischaemia first have a fasciotomy, and if there are more than two compartments which are non-viable, they have a primary amputation, whilst the rest undergo popliteal revascularisation. Patients with clearly non-salvageable Rutherford 3 limbs do not undergo a CT angiogram unless there are other indications and have a fasciotomy to document non-viable muscles prior to amputation. Patients with fractures or dislocations have external fixators applied after revascularisation unless reducing the bony segment first facilitates better exposure of the injured vessel.

All statistical tests were performed using STATA/SE 17.0. Categorical data were described by means of frequencies, and percentages. The Shapiro-Wilk test and graphical methods (histograms and normal probability plots) were utilised to carry out tests for normality. Medians and interquartile ranges (IQRs) were utilised to describe the data.

The significance level was set at a *P* < 0.05. Sensitivities and specificities of the MESS and POPSAVEIT scoring systems were also reported for the outcome variables of amputation and mortality.

We obtained ethical approval from the Gauteng Health Research Committee as well as from the Wits (University of Witwatersrand) Human Research Ethics Committee (HREC).

## Results

Sixty-four patients with a PAI were included in the study, of which 52 (81.3%) were male and 54 (84.4%) were under the age of 50 (Table 1). Of these, 41 (64.1%) had isolated limb injuries and notably, 1 patient died prior to any surgical intervention and was included as a mortality.

**Table 1:** Demographic data and clinical characteristics in amputation cohort compared to limb salvage cohort

	Amputation (*n = *23)	Limb Salvage (*n = *40)	*P value*
Age (median and IQR)	30 (26–37)	32 (27-47.5)	0.41
< 30 (n in years)	13 (44.8%)	16 (55.2%)	0.44
31–50	7 (29.2%)	17 (70.8%)	
> 50	3 (30.0%)	7 (70.0%)	
Sex (male/female)	19/4	32/8	> 0.99
Mechanism of Injury (%)			
Blunt trauma	12 (52.2%)	18 (45.0%)	0.58
Penetrating trauma	11 (47.8%)	22 (55.0%)	
Time delay (from injury to surgery)			
Total delay in minutes	680 (380–880)	707.5 (325-1222.5)	
Delay > 6 h (%)	19 (82.6%)	28 (70.0%)	0.37
Delay > 11 h (%)	11 (47.8%)	21 (52.5%)	0.80
Associated Injuries			
Knee dislocations	3 (13.0%)	10 (25.0%)	0.34
Lower limb fractures	18 (78.3%)	30 (75.0%)	> 0.99
Tibial plateau fracture	4 (17.4%)	10 (25.0%)	0.55
Severity Scoring Systems			
High Risk POPSAVEIT (≥3)	17 (73.9%)	24 (60.0%)	0.29
High Risk MESS (≥7)	14 (60.9%)	17 (42.5%)	0.20
High Risk Both Scoring Systems	11 (47.8%)	13 (32.5%)	0.29

IQR = interquartile range, FFH = Fall from height, GSW = Gunshot wound, MVA = Motor vehicle accident, PVA = Pedestrian vehicle accident, MBC = Motorbike collision, POPSAVEIT = Popliteal scoring assessment for vascular extremity injuries in trauma, MESS = Mangled extremity severity score

**Fig. 2:**
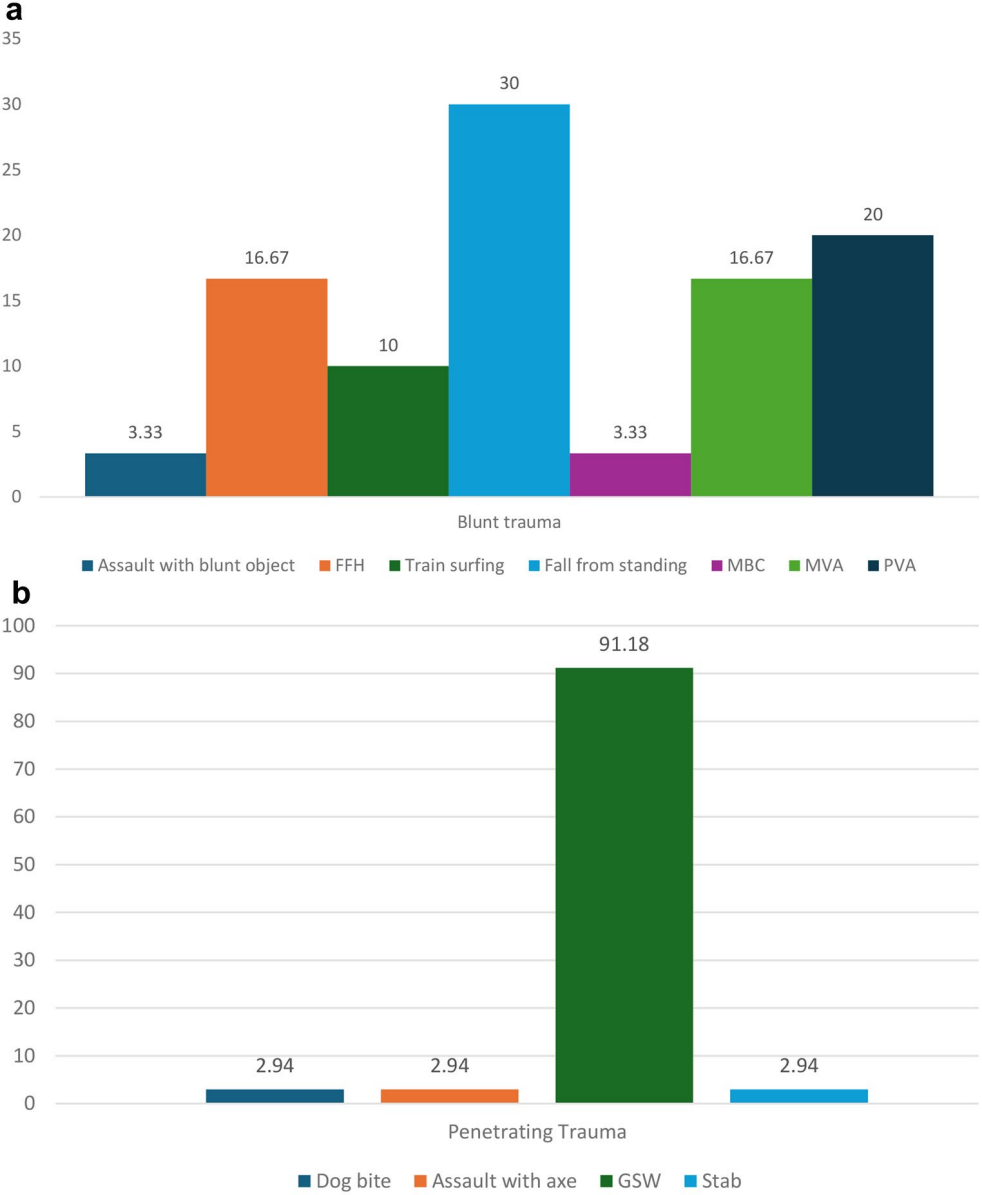
**a** Blunt mechanism of injury by categories. **b**: Penetrating mechanism of injury by categories

Blunt PAIs accounted for 30 (46.9%) of the PAIs whilst penetrating injuries resulted in 34 (53.1%) injuries (Fig. 2a, b). Forty-eight (75.0%) of our patients had associated fractures, and of these fractures, 14 (21.9%) were tibial plateau fractures and 13 (20.3%) had associated knee dislocations (Table 1). Fifty-six patients (87.5%) had absent pulses on clinical examination. Furthermore, 17 of the patients (26.6%) had absent doppler signals in the emergency department. Computer tomography (CT) scans were performed in 50 (78.1%) patients.

Forty-seven (73.4%) had a delay from time of injury to time of surgery of more than 6 h. The median delay from time of injury to time of arrival at the emergency department was 478 min (IQR = 70–625). The median time from arrival at hospital to time of surgery was 368 min (IQR = 127.5–565).

In the group of patients who had a delay to surgery of more than 6 h, 19 (40.4%) patients had eventual amputations. The rate of amputation amongst the 23 patients who were operated within 6 h was 17.4%, however this was not a statistically significant difference (*p* = 0.371).

Eighteen (28.6%) patients underwent a primary amputation whilst 45 (71.4%) had a revascularization attempt using Reverse Saphenous Vein Graft (RSVG). No other surgical conduits (such as polytetrafluoroethylene (PTFE) grafts, primary repairs, or temporary shunts) were utilised.

In the group of patients with blunt trauma 40.0% had amputations whilst in the penetrating group there were 33.3%, but we found no statistically significant difference between the two (*p* = 0.611). Thirty-four patients had penetrating injuries. Of these 8 (23.5%) had a primary amputation, and 22 had a RSVG interposition, whilst 3 patients required a delayed amputation after an initial revascularization and fasciotomy. Thus, 11/33 (33.3%) had amputations. The three patients who sustained injuries by falling from a moving train, had eventual amputations.

Those with knee dislocations secondary to blunt force trauma, 13 patients, 3 (23.1%) had amputations. In patients with concomitant fractures and an arterial injury, 18 (37.5%) had amputations and 4 (28.6%) of the patients with tibial plateau fractures eventually underwent amputations. Again, no statistical difference was found in those with these associated injuries and those without, in terms of amputation risk.

Of the 45 patients who underwent a RSVG revascularisation, 5 (11.1%) had to undergo a secondary amputation. Thus 40/63 (63.5%) had successful limb salvage and the amputation rate was 36.5% (23/63).

In the blunt trauma group, there were no mortalities compared with the penetrating trauma group where there were 4 mortalities (*p* = 0.116). The mortality rate was 6.3%. All the mortalities had surgery within 6 h of their injuries, and none had a popliteal artery related mortality.

In the POPSAVEIT score (Table 2), a score of ≥3 is considered high risk. Forty-one (64.1%) patients had a score of ≥3, indicating a strong likelihood of amputation, however, only 23 (36.5%) patients in total went on to have an amputation, 17 (73.9%) of those being in the high-risk categories, and 6 (26.1%) being considered of low risk. A MESS, score of ≥7 is considered high risk for amputation, and 31 (49.2%) patients had a high-risk score in this study, and again, in the high-risk category 14 (22.2%) had actual amputations (Table 3). In our cohort, neither the POPSAVEIT nor the MESS scores were accurate predictors of limb salvage (Table 4).

**Table 2:** POPSAVEIT scores of study patients

Score	Amputation *n* = 23 (%)	Limb Salvage *n* = 40 (%)
0	0 (0)	3 (7.5)
1	2 (8.7)	7 (17.5)
2	4 (17.4)	6 (15.0)
3	11 (47.8)	15 (37.5)
4	6 (26.1)	8 (20.0)
5	0 (0)	1 (2.5)

**Table 3:** MESS of study patients

Score	Amputation *n* = 23 (%)	Limb Salvage *n* = 40 (%)
2	0 (0)	1 (2.5)
3	1 (4.4)	3 (7.5)
4	2 (8.7)	3 (7.5)
5	2 (8.7)	9 (22.5)
6	4 (17.4)	7 (17.5)
7	3 (13.0)	8 (20.0)
8	7 (30.4)	5 (12.5)
9	0 (0)	2 (5.0)
10	3 (13.0)	1 (2.5)
11	1 (4.4)	1 (2.5)

**Table 4:** Comparison of MESS and POPSAVEIT Scoring Systems

Parameter	Amputation	Mortality
**POPSAVEIT High Risk ≥ 3**		
Rate	17 (41.46%)	2 (4.88%)
Sensitivity	73.9%	50.0%
Specificity	40.0%	35.0%
PPV	41.5%	4.9%
NPV	72.7%	91.3%
**MESS High Risk ≥ 7**		
Rate	14 (45.16%)	1 (3.23%)
Sensitivity	60.9%	25.0%
Specificity	57.5%	50.0%
PPV	45.2%	3.2%
NPV	71.9%	90.9%

## Discussion

Popliteal artery injuries though uncommon are associated with high rates of amputation. Amputation rates for civilian popliteal injuries have previously been reported to be between 14.5% and 25% and for the military population, it was found to be up to 30% [17]. In patients who have successful limb salvage, often concomitant orthopaedic injuries, soft tissue injuries and the need for muscle debridement prolong rehabilitation and return to full ambulatory function. Furthermore, PAIs place a significant burden on scarce resources which is more pronounced in resource poor health systems.

Outcomes in PAIs are determined by a composite of inputs including systems factors and patient factors. The system factors include pre-hospital referral pathways, in-hospital triage systems, and technical surgeon competencies. Patient related factors include the mechanism of injury (MOI), other associated injuries, and patient co-morbidities. As such, ours is an important study as it aimed to define the various factors with a view to improving outcomes.

We found a primary amputation rate of 28.6% (18 patients) and this was within the context of 73% (46 patients) presenting after 6 h. In a large retrospective series (164 patients) from Cape Town, Banderker et al. described a low primary amputation rate of 13.9% but an overall amputation rate of 37.5% [10], and a smaller series from the West Indies had an overall amputation rate of 28% [18]. Our overall amputation rate was 36% and this was in keeping with the Cape Town Study. A contemporary large study in America reports a 16% amputation rate [15].

In our study, only 23 patients arrived at the hospital within 6 h, and their amputation rate was 17.4% which is in-line with contemporary literature from first world countries, whereas in ours and the Cape Town experience, most patients arrived and were operated on after 6 h (mean: 14 h in our study). Thus, modifiable outcomes in popliteal injuries are largely determined by pre-hospital factors, and this is where the focus in our environment should be. Awareness of possible popliteal artery injuries within the 1st responder communities needs to be increased and triage systems require the inclusion of PAIs as ‘P1’ injuries requiring immediate transport to the appropriate trauma centres. A mechanism of injury consistent with a PAI in combination with no pulse should be referred directly to the appropriate centre.

In addition to patients presenting late, we found major delays to theatre even after arrival to hospital. Thus, our in-hospital triage protocols require revisiting and patients with confirmed popliteal injuries should be prioritised emergently.

There are however some positives to take home in our results. Of the 47 patients who had a delayed presentation to hospital (> 6 h), 28 (60%) had successful limb salvage. Our policy is to offer all patients a fasciotomy (even those with no motor or sensation), and in patients who have only 2 non-viable compartments (of 4), to offer them a trial of revascularisation and relook their muscle compartments after 48 h. In patients with 3 or all compartments non-viable on fasciotomy, we perform a primary amputation. Even though the delay to revascularisation was long in our patient cohort, in those who had a delayed revascularisation very few required a secondary amputation (11%). Additionally, all patients had a RSVG. The vascular surgical literature is overwhelmingly in support of a single segment RSVG in below knee revascularisations as it has better patency [19], and our data demonstrates that even in a trauma cohort a saphenous vein graft should be sought, and can be found in most patients. Prior to conducting the study, we anticipated many more penetrating injuries, however this was not our finding. The mechanism of injury, nor soft tissue or bone injuries predicted limb loss, and it appears that ischaemia as the common factor is the major determinant of limb salvage.

Furthermore, we found that the common risk prediction scores for popliteal injuries were inaccurate in our setting as they were not validated in environments with such a long delay to revascularisation. Both the MESS and the POPSAVEIT score were poor in predicting an amputation. The POPSAVEIT score was a multi-centre study including 355 patients where a score of more than 3 predicted an amputation [15]. However, in this cohort only 33% of patients were revascularized after 6 h, whereas in our study 73.4% had a delay to surgery. In our cohort, 41 (64.1%) patients had a score of ≥3, indicating a strong likelihood of amputation, however, only 23 (36.5%) patients with a score of ≥3 went on to have an amputation. Seventeen of these being in the high-risk category, and six being considered of low risk. More concerning than the positive prediction for an amputation through the POPSAVEIT score, was lack of reassurance from a low-risk score as six low-risk patients lost their limbs. Similarly, the MESS score had both a poor positive and negative predictive value in our setting. Thus, scoring systems may predict patients at high risk but their clinical applicability cannot be relied upon solely, and our policy of muscle assessment through a fasciotomy should be considered the gold standard and cannot be replaced by scoring systems.

## Limitations

Ours was a retrospective study and relied upon findings documented at the time of presentation. Also, we described only short-term outcomes and were unable to describe how many patients returned to function in the longer term.

## Conclusion

PAIs injuries occur predominately in young males, and in LMICs where manual labour is a means to income for most, limb salvage is even more critical. In HICs, rates of amputation have decreased to approximately 16–20%, largely due to the decrease in time of revascularisation. Pre-hospital systems require strengthening in order to save limbs.

Fasciotomies and muscle assessment should be attempted in all patients with marginal features of limb viability, as limb salvage rates are still acceptable in this cohort and common scoring systems should not be relied on, more so in patients with a delay to revascularisation.

## Data Availability

The data that supports the findings of this study are not openly available due to reasons of sensitivity and are available from the corresponding author upon request.
